# Explainable machine learning model for predicting furosemide responsiveness in patients with oliguric acute kidney injury

**DOI:** 10.1080/0886022X.2022.2151468

**Published:** 2023-01-16

**Authors:** Meng Jiang, Chun-qiu Pan, Jian Li, Li-gang Xu, Chang-li Li

**Affiliations:** a Emergency and Trauma Center, The First Affiliated Hospital, Zhejiang University School of Medicine, Hangzhou, China; b Department of Emergency Medicine, Nanfang Hospital, Southern Medical University, Guangzhou, China; c Department of Traumatic Surgery, Tongji Hospital, Tongji Medical College, Huazhong University of Science and Technology, Wuhan, China; d Department of Critical Care Medicine, Wuhan Central Hospital, Tongji Medical College, Huazhong University of Science and Technology, Wuhan, China; e Department of FSTC Clinic of The First Affiliated Hospital, Zhejiang University School of Medicine, Hangzhou, China

**Keywords:** Oliguric acute kidney injury, furosemide responsiveness, machine learning, XGBoost modeling

## Abstract

**Background:**

Although current guidelines didn’t support the routine use of furosemide in oliguric acute kidney injury (AKI) management, some patients may benefit from furosemide administration at an early stage. We aimed to develop an explainable machine learning (ML) model to differentiate between furosemide-responsive (FR) and furosemide-unresponsive (FU) oliguric AKI.

**Methods:**

From Medical Information Mart for Intensive Care-IV (MIMIC-IV) and eICU Collaborative Research Database (eICU-CRD), oliguric AKI patients with urine output (UO) < 0.5 ml/kg/h for the first 6 h after ICU admission and furosemide infusion ≥ 40 mg in the following 6 h were retrospectively selected. The MIMIC-IV cohort was used in training a XGBoost model to predict UO > 0.65 ml/kg/h during 6–24 h succeeding the initial 6 h for assessing oliguria, and it was validated in the eICU-CRD cohort. We compared the predictive performance of the XGBoost model with the traditional logistic regression and other ML models.

**Results:**

6897 patients were included in the MIMIC-IV training cohort, with 2235 patients in the eICU-CRD validation cohort. The XGBoost model showed an AUC of 0.97 (95% CI: 0.96–0.98) for differentiating FR and FU oliguric AKI. It outperformed the logistic regression and other ML models in correctly predicting furosemide diuretic response, achieved 92.43% sensitivity (95% CI: 90.88–93.73%) and 95.12% specificity (95% CI: 93.51–96.3%).

**Conclusion:**

A boosted ensemble algorithm can be used to accurately differentiate between patients who would and would not respond to furosemide in oliguric AKI. By making the model explainable, clinicians would be able to better understand the reasoning behind the prediction outcome and make individualized treatment.

## Introduction

Acute kidney injury (AKI) is a common syndrome of acute renal function impairment in critically ill patients, which carries a high morbidity and mortality rate [1]. According to the Kidney Disease: Improving Global Outcomes (KDIGO) guidelines [2], AKI can be defined by either a reduced urine output (UO) or an elevation in serum creatinine (SCr). In clinical practice, loop diuretics are often applied to prevent AKI by increasing the UO [3,4]. As the most common loop diuretic, furosemide is widely used in critically ill patients, and numerous clinical trials have been conducted to assess its effectiveness in AKI. In some studies, furosemide was found to be associated with neutral or deleterious effects in AKI [4–7]. In contrast, Zhao et al. [8] found that furosemide was associated with improved short-term survival and recovery of renal function in critically ill patients with AKI, especially in patients with AKI UO stage 2–3 degree.

As oliguria is the main indication for furosemide administration in clinical scenario, oliguric AKI should be considered as a single phenotype in assessing the effect of furosemide. It has been noticed that furosemide was associated with poor outcomes in patients with high SCr level (3.8 mg/dL [5], or 3.3 mg/dL [9]) or AKI stage 2–3 according to SCr criteria [8], while it imposed insignificant effects in patients with mild AKI level (SCr, 1.8 mg/dL [10]). These results implied that the curative effect of furosemide on AKI may be influenced by the increased level of SCr, or to say, affected by the severity of kidney injury.

Patients who develop AKI often need renal replacement therapy (RRT), but the optimal timing for RRT is controversial. For oliguric AKI, furosemide-induced UO increase reflects the integrity of renal tubular function and may reduce the chance for invasive RRT procedure. However, lack of response to furosemide and the delaying RRT procedure in the course of AKI would subject patients to adverse consequences [11]. Thus, it’s of clinical importance to identify the patients who will benefit from furosemide at an early stage. Currently, there is no effect clinical tool to distinguish oliguric AKI patients who are furosemide-responsive (FR) and furosemide-unresponsive (FU) in terms of UO response.

To analyze numerous variables that may be associated with furosemide responsiveness in oliguric AKI, an effective approach is required for the development of precise prediction model. Machine learning (ML) has been applied in areas of critical care medicine such as outcome prediction, diagnosis assessment, and treatment decision-making [12–17]. In this study, we used a boosted ensemble algorithm (XGBoost) to predict the furosemide responsiveness in patients with oliguric AKI. Since one of the limitations for ‘black-box’ ML model in a practical setting is the lack of physician trust, we used interpretable ML method, called SHapley Additive exPlanations (SHAP) [18–20], to provide insight into how the prediction was made.

## Methods

### Study cohort

We conducted this retrospective study based on two large US-based critical care databases named Medical Information Mart for Intensive Care-IV (MIMIC-IV) [21] and eICU Collaborative Research Database (eICU-CRD) [22]. The MIMIC-IV contains comprehensive and high-quality data of 524,520 admissions (including 257,366 patients) admitted to intensive care units (ICUs) at the Beth Israel Deaconess Medical Center during 2008–2019. The eICU-CRD covered 200,859 ICU admissions (including 139,367 patients) between 2014 and 2015 at 208 US hospitals. Since the study was an analysis of publicly available database with preexisting institutional review board (IRB) approval, IRB approval was exempted by our institution. The study was reported according to the recommendations of the Transparent Reporting of a multivariable prediction model for Individual Prognosis or Diagnosis (TRIPOD) statement [23].

### Associated definitions

The training cohort was defined as data set used for model construction, while validation cohort was defined as data set used for model assessment. The metrics used for evaluating our model including area under the receiver-operating characteristic curve (AUC), accuracy, sensitivity, specificity, positive predictive value (PPV), and negative predictive value (NPV). The details on XGBoost, support vector machines (SVM), K-Nearest Neighbor (KNN), and random forest are described in the Supplementary Material.

### Participants

**Figure 1.**
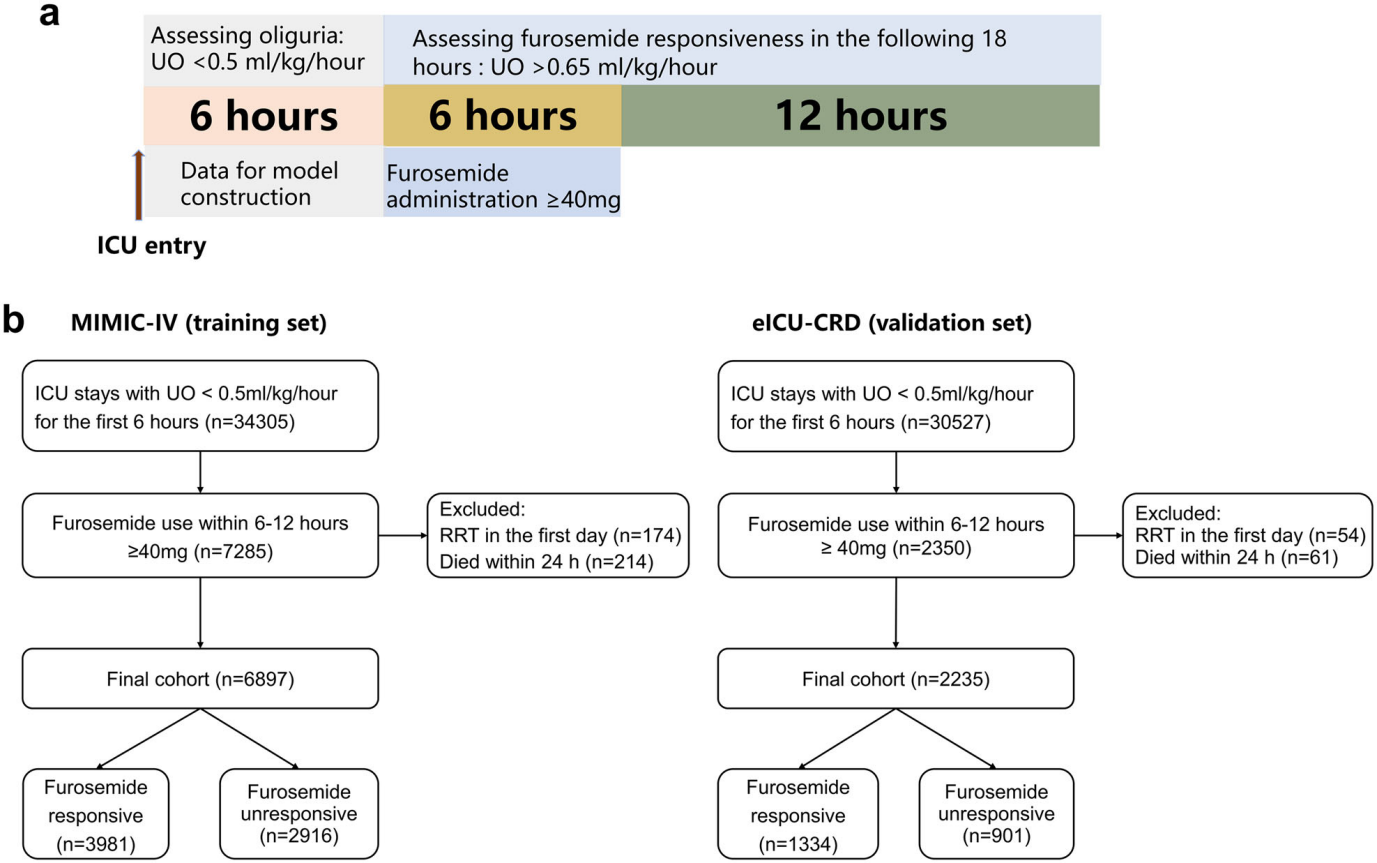
Design and patient screening of the study. (a) Schematic illustration of the time windows for the definition of oliguria and furosemide responsiveness. Oliguria was defined as urine output < 0.5 mL/kg/h for the first 6 h after ICU admission. Diuretic responsiveness was defined as urine output > 0.65 mL/kg/h for 18 h after administration of furosemide. It is noted that the time window for the definition of oliguria preceded the furosemide treatment. (b) Flow chart of patient selection.

Patient was considered eligible for inclusion when UO < 0.5 mL/kg/h for the first 6 h after ICU admission. This criterion was consistent with the UO component of the KDIGO definition [2]. To examine the impact of furosemide on subsequent diuretic response, only patients with furosemide administration ≥ 40 mg (*via* intravenous pathway) within 6–12 h following the initial 6 h for assessing oliguria were eligible (Figure 1(a)). Since fluid intake can also affect the UO, we calculated the total intake fluid volume during 6–24 h for bias assessment. Fluid intake and UO were extracted from the nursing chart system. Patients died within 24 h after ICU admission, received any other diuretics and/or RRT on day 1, and had received diuretics within 24 h prior to ICU admission were excluded.

### Outcome (furosemide responsiveness)

The UO during 6–24 h following the initial 6 h for determine oliguria was used as the outcome (Figure 1(a)). Patients were considered as FR AKI if he/she had UO greater than 0.65 mL/kg/h after the first 6 h, equivalent to a 30% increase compared with the baseline value, according to thresholds reported in previous study [13]. Otherwise, they were defined as FU AKI.

### Predictors of FR AKI

The clinical and laboratory variables within the first 6 h of ICU admission were extracted from the MIMIC-IV and eICU-CRD database. For laboratory variables and vital signs that with multiple measurements, both the minimum and maximum values were evaluated. Age, gender, comorbid illness, ethnicity, admission type, vasopressor use, mechanical ventilation, presence of infection, and vital signs including respiratory rate, blood pressure, heart rate, Spo2, and temperature were analyzed. In addition, laboratory data including SCr, blood urea nitrogen (BUN), glucose, lactate, bicarbonate, serum electrolytes (chloride, potassium, sodium), routine blood tests (white blood cell count [WBC], hematocrit, hemoglobin, platelet), coagulation profile (PT, aPTT, INR), PaCO2, and pH were included.

### Data pre-processing

In the present study, albumin, alanine aminotransferase (ALT), aspartate aminotransferase (AST), and bilirubin that with more than 60% missing values were excluded from further analysis. All other variables had less than 20% missing values, and single imputation with regression method was used to impute the missing values [24]. The distribution of missing value is provided in Table S1. Categorical and ordinal variables were transferred to numerical variables based on their clinical significance. The XGBoost model was preferred over the other ML models in this study because of its ability to deal with missing data. For the XGBoost model, no imputation was performed for both the training and validation cohort. The single imputation for missing value was only performed for other ML models for comparison.

### Model development

The MIMIC-IV cohort was used to develop the XGBoost model, using an open-source XGBoost library. XGBoost is a non-parametric model that uses an ensemble of gradient-boosted decision trees to draw a parallel between boosting and gradient descent in function space [19]. The advantage of applying a boosted ensemble algorithm is that it combines multiple weak classifiers to generate a single strong classifier, which can greatly improve prediction modeling. XGBoost algorithm includes more than 20 hyperparameters such as ‘Learning rate’, ‘Maximum depth’, ‘Min child weight’, and ‘Number estimators’, et al. Hyperparameter optimization and cross-validation were conducted through GridSearchCV strategy [25,26] to prevent overfitting and increase the model’s accuracy.

Although ML methods can achieve better prediction performance compared with traditional scoring systems, the ‘black-box’ pattern are usually unexplainable and clinicians are reluctant to accept them in clinical practice [27,28]. For this reason, we used the SHAP method to explain the output of the XGBoost model. The SHAP method assigns an importance value to each variable for a particular prediction, and it was used to determine the impact of a particular variable on furosemide responsiveness prediction, as well as the direction of this association.

### Model evaluation

We also fitted several other models including logistic regression, support vector machines (SVM), K-Nearest Neighbor (KNN), and random forest for comparison. The AUC, accuracy, sensitivity, specificity, PPV, and NPV of these models were compared with the XGBoost model. For modeling, the MIMIC-IV cohort was used as the training set, then the model was validated in the independent eICU-CRD cohort.

### Statistical analysis

Data were analyzed with R version 3.5.0 (RStudio) and Python 3.8 (Python Software Foundation). Continuous variables were expressed as median (interquartile range) or mean (standard derivation) according to the data distribution, whereas categorical variables were expressed as count and percent. Differences between continuous and categorical parameters were analyzed using Wilcoxon rank sum test, chi-square test, or Fisher exact test as appropriate. Different AUCs were compared with Delong test. The hazard ratio (HR) for survival difference between furosemide responsive and furosemide unresponsive groups was calculated by univariate cox regression and Kaplan Meier analysis. All tests were two-sided and performed at the 0.05 significance threshold. The major packages of R and python software used in this study were provided in the (Table S2). The code for MIMIC database can be obtained at: https://github.com/MIT-LCP/mimic-code/; the code for eICU-CRD can be obtained at https://github.com/mit-lcp/eicu-code.

## Results

### Patient characteristics

In the MIMIC-IV cohort, of the 34,305 patients with UO < 0.5 mL/kg/h for the initial 6 h after ICU admission, 7285 patients (21.2%) received furosemide administration ≥ 40 mg within the following 6 h. A number of 174 patients were excluded because they received RRT on the first day. 214 patients died within 24 h after ICU admission. Finally, a total of 6897 patients were included in our analysis for model construction; 3981 patients had FR AKI, while 2916 patients had FU AKI on day 1 in ICU (Figure 1(b)). Following the same procedure, 2235 patients (1334 FR and 901 FU) with oliguric AKI were included from the eICU-CRD database for model validation. Clinical characteristics of the two cohorts of patients are listed in Table 1 and Table S3.

**Table 1.** Patient characteristics and clinical variables.

Variables	Training set (MIMIC-IV)				Validation set (eICU-CRD)					
	Unresponsive	Responsive	^a^*p* Value	^a^SMD	Unresponsive	Responsive	^a^*p* Value	^a^SMD	^b^*p* Value	^b^SMD
Patient population, n	2916	3981			901	1334				
Age (SD)	70.1 (12.8)	70.0 (13.1)	.736	0.008	70.4 (13.1)	70.5 (13.0)	.935	0.003	.167	0.034
Male (%)	1701 (58.3)	2323 (58.4)	1	<0.001	503 (55.8)	735 (55.1)	.767	0.015	.015	0.06
Comorbid illness										
Congestive heart failure (%)	1563 (53.6)	1775 (44.6)	<.001	0.25	189 (21.0)	229 (17.2)	.027	0.097	<.001	0.663
Chronic kidney disease (%)	997 (34.2)	840 (21.1)	<.001	0.181	257 (28.5)	262 (19.6)	<.001	0.209	.001	0.079
Severe liver disease (%)	146 (5.0)	104 (2.6)	<.001	0.296	66 (7.3)	68 (5.1)	.037	0.092	<.001	0.111
Total fluid intake between 6 and 24 h (median [IQR])	3340.0 [1400.0, 6100.0]	3350.0 [1380.0, 6300.0]	.794	0.006	3750.0 [1420.0, 7590.0]	3670.0 [1490.0, 7215.0]	.616	0.034	<.001	0.158
Total furosemide administration between 6 and 12 h (median [IQR])	60 [40, 100]	40 [40, 60]	<.001	0.25	50 [40, 80]	40 [40, 55]	<.001	0.101	.01	0.002
Length of ICU stay, day (SD)	5.4 (6.5)	5.0 (6.1)	.003	0.072	5.1 (6.2)	4.6 (5.4)	.057	0.081	.024	0.056
In hospital mortality (%)	441 (15.1)	400 (10.0)	<.001	0.153	124 (13.8)	115 (8.6)	<.001	0.164	.061	0.047
Ethnicity (%)			.16	0.047			.564	0.046	<.001	0.126
African American	244 (8.4)	304 (7.6)			63 (7.0)	85 (6.4)				
White	2124 (72.8)	2863 (71.9)			705 (78.2)	1032 (77.4)				
Other	548 (18.8)	814 (20.4)			133 (14.8)	217 (16.3)				
Admission type (%)			<.001	0.137			.19	0.079	<.001	0.786
Elective	707 (24.2)	1161 (29.2)			245 (27.2)	400 (30.0)				
Emergency	1480 (50.8)	1764 (44.3)			647 (71.8)	914 (68.5)				
Urgent	729 (25.0)	1056 (26.5)			9 (1.0)	20 (1.5)				
Vasopressor use, n (%)	1081 (37.1)	1437 (36.1)	.421	0.02	304 (33.7)	445 (33.4)	.887	0.008	.011	0.063
Infection, n (%)	777 (26.6)	1102 (27.7)	.354	0.023	444 (49.3)	569 (42.7)	.002	0.133	<.001	0.383
Mechanical ventilation, n (%)	1163 (39.9)	1518 (38.1)	.147	0.036	454 (50.4)	722 (54.1)	.091	0.075	<.001	0.279
Minimum bicarbonate (mmol/L, median [IQR])	23.0 [20.0, 25.0]	23.0 [21.0, 25.0]	<.001	0.177	24.0 [21.0, 26.0]	24.0 [22.0, 26.2]	.001	0.133	<.001	0.211
Maximum bicarbonate (mmol/L, median [IQR])	23.0 [21.0, 26.0]	24.0 [22.0, 26.0]	<.001	0.151	24.0 [22.0, 27.0]	25.0 [22.0, 27.0]	.007	0.091	<.001	0.243
Minimum creatinine (mg/dL, median [IQR])	1.2 [0.9, 1.9]	0.9 [0.7, 1.2]	<.001	0.478	1.1 [0.8, 1.8]	0.9 [0.7, 1.3]	<.001	0.406	.229	0.039
Maximum creatinine (mg/dL, median [IQR])	1.2 [0.9, 1.9]	0.9 [0.7, 1.3]	<.001	0.481	1.2 [0.9, 1.8]	1.0 [0.8, 1.3]	<.001	0.407	.367	0.023
Minimum chloride (mmol/L)	101.7 (6.0)	102.2 (5.5)	<.001	0.091	101.5 (6.0)	102.0 (5.2)	.021	0.1	.19	0.033
Maximum chloride (mmol/L)	104.7 (6.9)	105.9 (6.6)	<.001	0.178	105.5 (6.9)	106.5 (6.6)	.001	0.141	<.001	0.107
Minimum glucose (mg/dL, median [IQR])	125.0 [106.0, 158.0]	121.0 [104.0, 145.0]	<.001	0.127	122.0 [105.0, 151.8]	117.0 [101.0, 140.0]	.001	0.135	<.001	0.057
Maximum glucose (mg/dL, median [IQR])	169.0 [134.0, 205.0]	164.0 [136.0, 196.0]	.035	0.057	162.0 [131.0, 196.8]	161.0 [134.0, 190.0]	.5	0.051	.001	0.07
Minimum hematocrit (%)	28.7 (6.9)	28.1 (6.5)	<.001	0.1	28.4 (6.8)	28.1 (6.9)	.278	0.048	.337	0.024
Maximum hematocrit (%)	34.3 (6.3)	35.2 (6.2)	<.001	0.15	34.5 (6.0)	35.3 (5.8)	.003	0.133	.482	0.018
Minimum hemoglobin (g/dL)	9.4 (2.2)	9.3 (2.1)	.021	0.06	9.4 (2.2)	9.4 (2.2)	.601	0.023	.263	0.028
Maximum hemoglobin (g/dL)	11.2 (2.2)	11.6 (2.1)	<.001	0.199	11.4 (2.0)	11.7 (2.0)	<.001	0.174	.023	0.059
Minimum lactate (mmol/L)	1.8 (1.5)	1.6 (0.9)	<.001	0.212	1.7 (1.5)	1.5 (0.8)	<.001	0.185	.005	0.077
Maximum lactate (mmol/L)	6.3 (7.2)	6.0 (7.1)	.094	0.041	6.1 (7.0)	5.9 (7.1)	.613	0.022	.47	0.018
Minimum platelet (×10^9^/L, median [IQR])	165.0 [121.0, 224.0]	155.0 [116.0, 210.0]	<.001	0.077	182.0 [136.0, 243.0]	174.0 [129.8, 236.0]	.038	0.066	<.001	0.203
Maximum platelet (×10^9^/L, median [IQR])	182.5 [137.0, 241.0]	174.0 [135.0, 228.0]	.002	0.064	205.0 [156.0, 268.0]	195.0 [152.0, 258.0]	.029	0.063	<.001	0.233
Minimum potassium (mmol/L)	4.1 (0.7)	3.9 (0.6)	<.001	0.295	4.1 (0.6)	3.9 (0.5)	<.001	0.271	.006	0.069
Maximum potassium (mmol/L)	4.9 (0.9)	4.9 (0.9)	.725	0.009	4.9 (1.0)	4.9 (1.0)	.238	0.052	.061	0.046
Minimum aPTT (s)	37.0 (20.2)	35.0 (18.3)	<.001	0.1	35.6 (16.3)	35.1 (17.8)	.509	0.031	.253	0.031
Maximum aPTT (s)	43.4 (26.8)	41.3 (24.4)	.002	0.084	42.5 (25.6)	41.1 (24.4)	.254	0.053	.424	0.021
Minimum INR	1.5 (1.0)	1.4 (0.7)	<.001	0.138	1.6 (1.1)	1.5 (0.8)	.02	0.105	.08	0.045
Maximum INR	1.7 (1.0)	1.6 (0.9)	<.001	0.1	1.7 (1.3)	1.6 (0.9)	.003	0.131	.125	0.039
Minimum PT (s)	16.8 (9.9)	15.5 (7.1)	<.001	0.143	17.1 (8.9)	16.2 (7.0)	.014	0.111	.027	0.059
Maximum PT (s)	18.2 (10.4)	17.2 (9.1)	<.001	0.098	18.8 (11.6)	17.5 (8.5)	.005	0.126	.134	0.039
Minimum sodium (mmol/L)	135.9 (4.8)	135.9 (4.5)	.835	0.005	136.2 (4.8)	136.4 (4.5)	.296	0.046	<.001	0.099
Maximum sodium (mmol/L)	138.2 (4.8)	138.7 (4.2)	<.001	0.101	138.8 (4.8)	139.2 (4.3)	.034	0.092	<.001	0.123
Minimum BUN (mg/dl, median [IQR])	24.0 [17.0, 41.0]	19.0 [14.0, 28.0]	<.001	0.4	24.0 [17.0, 41.0]	19.0 [14.0, 29.0]	<.001	0.359	.229	0.018
Maximum BUN (mg/dl, median [IQR])	25.0 [17.0, 42.0]	19.0 [14.0, 29.0]	<.001	0.401	25.0 [17.0, 43.0]	20.0 [15.0, 31.0]	<.001	0.36	.099	0.027
Minimum WBC (×10^9^/L)	12.5 (8.7)	11.8 (7.1)	.001	0.088	12.0 (9.8)	11.4 (5.8)	.06	0.08	.014	0.063
Maximum WBC (×10^9^/L)	19.3 (13.8)	19.2 (14.0)	.666	0.011	16.2 (11.4)	15.3 (10.8)	.071	0.077	<.001	0.281
Minimum heartrate (/min)	75.5 (16.5)	76.1 (15.1)	.109	0.039	75.5 (16.5)	76.7 (14.9)	.073	0.078	.319	0.025
Maximum heartrate (/min)	92.8 (19.8)	93.8 (18.3)	.044	0.05	91.8 (18.6)	94.1 (17.2)	.003	0.127	.687	0.01
Minimum systolic BP (mmHg)	95.2 (17.0)	98.0 (17.5)	<.001	0.162	96.5 (17.7)	98.9 (18.2)	.003	0.132	.012	0.062
Maximum systolic BP (mmHg D)	133.2 (21.6)	136.5 (21.5)	<.001	0.155	134.5 (21.5)	138.8 (22.2)	<.001	0.195	<.001	0.09
Minimum diastolic BP (mmHg)	49.0 (11.2)	51.0 (10.8)	<.001	0.179	48.3 (11.8)	49.9 (11.0)	.001	0.146	.001	0.08
Maximum diastolic BP (mmHg)	74.8 (18.9)	75.5 (17.0)	.102	0.04	73.4 (17.6)	74.8 (16.2)	.053	0.084	.025	0.057
Minimum mean BP (mmHg)	62.1 (12.9)	64.5 (12.5)	<.001	0.192	61.1 (14.4)	63.6 (12.7)	<.001	0.191	.007	0.066
Maximum mean BP (mmHg)	92.3 (22.9)	94.6 (20.3)	<.001	0.103	91.5 (22.3)	95.1 (22.6)	<.001	0.159	.918	0.003
Minimum respiratory rate (/min)	14.5 (4.3)	14.4 (4.4)	.641	0.012	14.2 (4.3)	14.2 (4.5)	.842	0.009	.026	0.055
Maximum respiratory rate (/min)	24.2 (6.6)	24.1 (6.9)	.292	0.026	23.7 (6.7)	23.9 (6.7)	.622	0.022	.041	0.051
Minimum temperature (°C)	36.3 (0.8)	36.3 (0.7)	.233	0.031	36.1 (0.9)	36.2 (0.8)	.125	0.072	<.001	0.16
Maximum temperature (°C)	36.8 (0.7)	36.8 (0.7)	.881	0.004	36.7 (0.8)	36.7 (0.8)	.321	0.047	.001	0.083
Minimum Spo2 (%)	94.1 (6.3)	94.7 (5.3)	<.001	0.103	94.1 (6.8)	94.5 (5.5)	.147	0.062	.58	0.014
Maximum Spo2 (%)	99.1 (2.0)	99.2 (1.6)	.002	0.077	99.1 (2.0)	99.1 (1.6)	.591	0.023	.42	0.02

*Notes:* Continuous variables were expressed as median (interquartile range) or mean (standard derivation) according to the data distribution, categorical variables were expressed as count and percent. SD: standard derivation; IQR: interquartile range; BP: blood pressure; BUN: blood urea nitrogen; SMD: standardized mean difference.

^a^Comparison between the furosemide responsive and furosemide unresponsive groups.

^b^Comparison between the training and validation groups.

In both the training and validation cohort, the total fluid intake during 6–24 h was comparable between the FU and FR groups (3340.0 [1400.0, 6100.0] vs 3350.0 [1380.0, 6300.0] ml for MIMIC-IV, *p* = .794; 3750.0 [1420.0, 7590.0] vs 3670.0 [1490.0, 7215.0] ml for eICU-CRD, *p* = .616). However, the FU group received higher dose of furosemide administration during this period (*p* < .001 for both cohort). The FU group had a significantly higher in hospital mortality than the FR group in both cohorts (15.1% vs 10.0% for MIMIC-IV, *p* < .001; 13.8% vs 8.6% for eICU-CRD, *p* < .001). The maximum SCr were higher in the FU group (1.2 [0.9, 1.9] vs 0.9 [0.7, 1.3] mg/dL for MIMIC-IV, *p* < .001; 1.2 [0.9, 1.8] vs 1.0 [0.8, 1.3] mg/dL for eICU-CRD, *p* < .001). Other details on clinical variables between FR and FU AKI groups are shown in Table 1.

### The stepwise logistic regression model

The results of backward stepwise logistic regression model are shown in Table S4. As expected, maximum SCr (odds ratio [OR] for each 0.1 mg/dL increase, 0.26; 95% confidence interval [CI], 0.11 to 0.60) was negatively associated with furosemide diuretic effect. On the contrary, a greater value of maximum hemoglobin (OR for each 1 g/dL increase, 1.34; 95% CI, 1.19 to 1.50), and maximum systolic BP (OR for each 1 mmHg increase, 1.01; 95% CI, 1.00 to 1.01) were associated with increased furosemide responsiveness. The strongest predictors for logistic regression model were maximum hemoglobin and maximum systolic BP (Figure S1).

### The XGBoost model

The major hyperparameters used in our analysis were listed in Table S5. Figure S2 shows the training process of the XGBoost model. As time goes by, the AUC of the validation set (Figure S2a, represented by the rising solid curve) achieved a final best AUC of 0.97 at the 500th round. The log-loss on the validation cohort is represented by the falling curve (Figure S2b). The similarity of the loss curves between the training and validation cohorts means that the model has no significant overfitting. The final assessment on the validation set was performed at round 500. Figure S3 gives the SHAP summary plot for the top 30 clinical features contributing to our XGBoost model’s prediction for furosemide responsiveness. According to the feature ranking, the maximal SCr was the most important variable to distinguish FR and FU AKI group. The SHAP dependence plot (Figure S4) can also facilitate understanding how a single feature affects the output of the model. We could figure out how the feature’s attributed importance changed as its values varied in the plot. SHAP values for specific variable exceeding zero represent an increased probability of furosemide responsiveness.

### Prediction performance on training and test set

**Figure 2.**
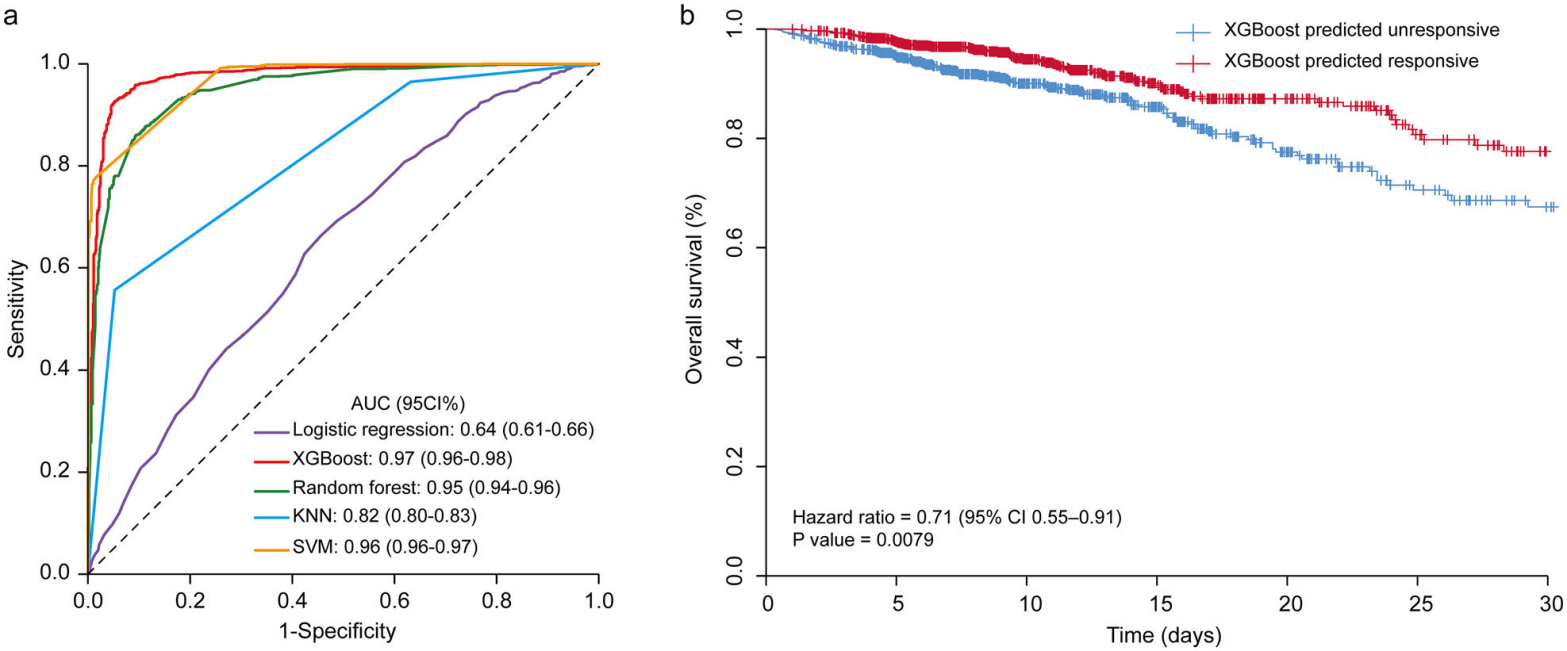
Performance of the XGBoost model. (a) ROC curves for XGBoost, logistic regression, random forest, KNN and SVM when predicting furosemide responsiveness. (b) Overall survival of patients relative to the furosemide responsiveness in the first 30-days. KNN: K-Nearest Neighbor; SVM: support vector machines.

Our model performed well in the training cohort (Table 2). In the external test set, the XGBoost model had a significantly greater AUC than the logistic regression (0.97 vs 0.64, *p* < .001 by Delong test) and KNN model (0.97 vs 0.82, *p* < .001) (Figure 2(a)), with good calibration (Figure S5). The precision recall curve and the net decision curves were shown in Figures S6 and S7. For sensitivity analysis, we constructed another XGBoost model based on the data after imputation. There is no significant deference between the two models (Figure S8). After considering the balanced performance of accuracy, sensitivity and specificity, the XGBoost model had the most powerful discrimination for predicting furosemide responsiveness (accuracy: 93.51%, sensitivity: 92.43%, specificity: 95.12%, PPV: 96.55%, NPV: 89.46%) (Table 2). The overall 30-day survival was significantly better in the group predicted as FR (HR 0.71, 95% CI 0.55–0.91; *p* = .0079) than the group predicted as FU (Figure 2(b)).

**Table 2.** Performance metrics of the machine learning models in the internal validation and test cohort.

a. Internal validation cohort								
	AUC	Accuracy	Sensitivity	Specificity	PPV	NPV	Brier score	Kappa^a^
Logistic regression	0.65 (0.62–0.67)	61.73% (59.58–63.84%)	64.97% (62.19–67.66%)	57.23% (53.85–60.54%)	67.83% (65.03–70.51%)	54.06% (50.77–57.32%)	0.236	0.22
XGBoost	0.99 (0.99–1.0)	99.85% (99.56–99.95%)	99.91% (99.51–99.98%)	99.76% (99.13–99.93%)	99.83% (99.38–99.95%)	99.88% (99.33–99.98%)	0.046	0.99
SVM	0.99 (0.99–1.0)	99.45% (99.02–99.69%)	99.66% (99.12–99.87%)	99.16% (98.23–99.59%)	99.40% (98.76–99.71%)	99.52% (98.77–99.81%)	0.073	0.98
KNN	0.69 (0.66–0.71)	64.58% (62.46–66.65%)	67.47% (64.72–70.10%)	60.57% (57.22–63.83%)	70.38% (67.63–72.98%)	57.29% (54.0–60.51%)	0.223	0.28
Random Forrest	0.99 (0.96–0.99)	98.05% (97.34–98.57%)	97.59% (96.54–98.33%)	98.69% (97.66–99.26%)	99.04% (98.29–99.46%)	96.72% (95.30–97.72%)	0.073	0.96

b. Test cohort								
	AUC	Accuracy	Sensitivity	Specificity	PPV	NPV	Brier score	Kappa^a^
Logistic regression	0.64 (0.61–0.66)	60.67% (58.63–62.68%)	62.74% (60.12–65.3%)	57.60% (54.35–60.79%)	68.66% (66–71.2%)	51.08% (48.01–54.15%)	0.225	0.20
XGBoost	0.97 (0.96–0.98)	93.51% (92.41–94.46%)	92.43% (90.88–93.73%)	95.12% (93.51–96.3%)	96.55% (95.41–97.42%)	89.46% (87.35–91.25%)	0.043	0.87
SVM	0.96 (0.96–0.97)	83.45% (81.85–84.93%)	99.85% (99.45–99.96%)	59.16% (55.91–62.32%)	78.35% (76.33–80.25%)	99.63% (98.65–99.9%)	0.092	0.63
KNN	0.82 (0.80–0.83)	71.41% (69.5–73.24%)	55.62% (52.94–58.27%)	94.78% (93.13–96.05%)	94.04% (92.17–95.49%)	59.06% (56.5–61.57%)	0.158	0.46
Random Forrest	0.95 (0.94–0.96)	88.1% (86.69–89.38%)	88.98% (87.19–90.55%)	86.79% (84.42–88.85%)	90.89% (89.21–92.33%)	84.18% (81.69–86.38%)	0.084	0.75

*Notes:* Unless otherwise indicated, data are percentages, with 95% confidence intervals in brackets. PPV: positive predictive value; NPV: negative predictive value.

^a^Measures the agreement between the prediction and the ground truth.

To further explore our model’s robustness, we repeated our analysis based on the dataset after removing all observations with missing data. The results are presented in Table S6.

### Model interpretation

**Figure 3.**
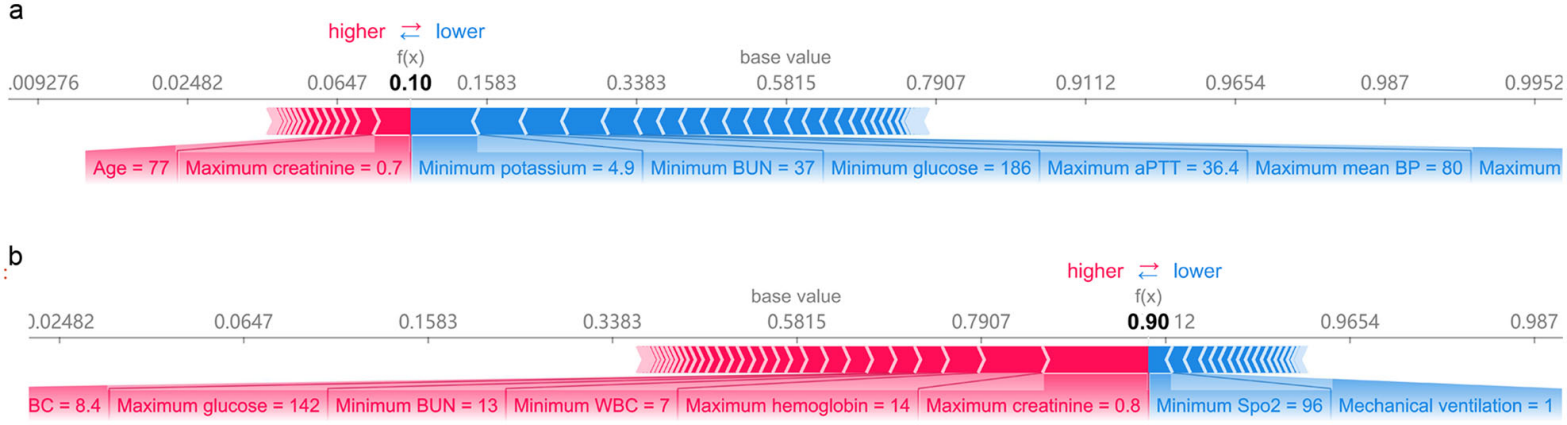
Explanation of the prediction results for specific instances. The base value (58.15%) is the average value of the furosemide responsiveness; the output values (heavy black) are the predicted diuretic response probability. (a) A low diuretic response probability that the features in blue push the value of the instance (calculated by the prediction model) lower than the average value. (b) A high diuretic response probability that the features in red push the value of the instance higher than the average value.

We employed SHAP method to explain how the trained XGBoost model makes prediction for specific patient. Figure 3(a,b) shows two such examples. The variables in red and blue bars represent positively effective factors and adversely effective factors for furosemide responsiveness prediction, respectively. Longer bars mean bigger variable importance. The heavy black value represents the predicted probability of furosemide responsiveness.

For the case in Figure 3(a), the patient (weighted 55 kg) received 80 mg furosemide and 1620 mL fluid during 6–24 h. The predicted probability for furosemide responsiveness was 10% compared with the baseline value of 58.15%; the oliguria continued to 24 h as predicted. Major factors detected by the model that increased the probability of furosemide responsiveness were age and maximal SCr. Major factors decreasing the furosemide responsiveness included minimum potassium and minimum BUN. For the instance in Figure 3(b), the patient (weighted 65.7 kg) received 40 mg furosemide and 2510 mL fluid during 6–24 h, the ML model predicted probability of furosemide responsiveness was 90%. The model successfully predicted the outcome, he achieved UO 2.37 mL/kg/h at 24 h.

## Discussion

We have explored the performance of advanced machine learning model in predicting the furosemide responsiveness in patients with oliguric AKI. With the rich ICU data collected from the MIMIC-IV and eICU-CRD databases, the XGBoost model showed excellent predicting power with a sensitivity of 92.43% and specificity of 95.12% for the detection of furosemide diuretic responsiveness. The model outperformed the conventional logistic regression and other ML models, which have important clinical implications.

An ability to accurately identify furosemide responsiveness in critically ill patients with oliguric AKI is clinically important to avoid ineffective furosemide therapy and the subsequent delaying RRT. Recent evidence suggested that delayed RRT increased mortality over the first 90 days [29]. Currently, there is no reliable method to distinguish between FR and FU oliguric AKI at an early stage. The ML model has a potential to help stratifying oliguric AKI patients immediately after ICU admission. Generally, the evidence so far available from clinical trials do not support furosemide as routine reliable AKI therapeutics [30]. However, AKI prevention or treatment by furosemide can be associated with favorable outcomes in certain situations [31,32], especially in patients with AKI UO stage 2–3 degree [8]. Just as Shen et al. stated that ‘Loop diuretic use in patients with AKI: different severity, different response’ [33].

It’s well established that higher SCr can result in attenuated response to furosemide in AKI patients [5,8,9], and this was confirmed by our ML model that elevated SCr level was the most important feature that affected the diuretic effect of furosemide. However, elevated SCr level can only reflect a relatively late stage of renal function impairment. An ensemble algorithm that integrated all available clinical data after ICU admission will provide more comprehensive information on the patients’ pathophysiological status, and hence more accurately reflect the severity of AKI.

Except for the severity of kidney injury per se, body volume status is another important factor that affects the furosemide responsiveness. A multicenter ICU study observed that, in patients with a higher fluid balance and a lower UO, diuretic use was associated with better survival [34]. Thus, the beneficial effects of furosemide on oliguric AKI may be mediated by optimization of volume status. We found that an increased minimum systolic BP within the first 6 h of ICU admission was an important predictor of FR (in both the XGBoost and logistic models). This was interpretable that minimum systolic BP has a direct relationship with the intravascular volume status. Conversely, a higher maximum heartrate on ICU admission might indicate hypovolemia, which was associated with a blunted diuretic response. By SHAP method, both the renal intrinsic damage biomarker (SCr) and volume state indicators (BP and heartrate) were identified as key predictors for furosemide responsiveness. This means that the XGBoost model could not only provide explainable predictions, but is also a safe tool in clinical practice, for pre-evaluation of hemodynamic status is the fundamental of safe administration of diuretics [30].

**Figure 4.**
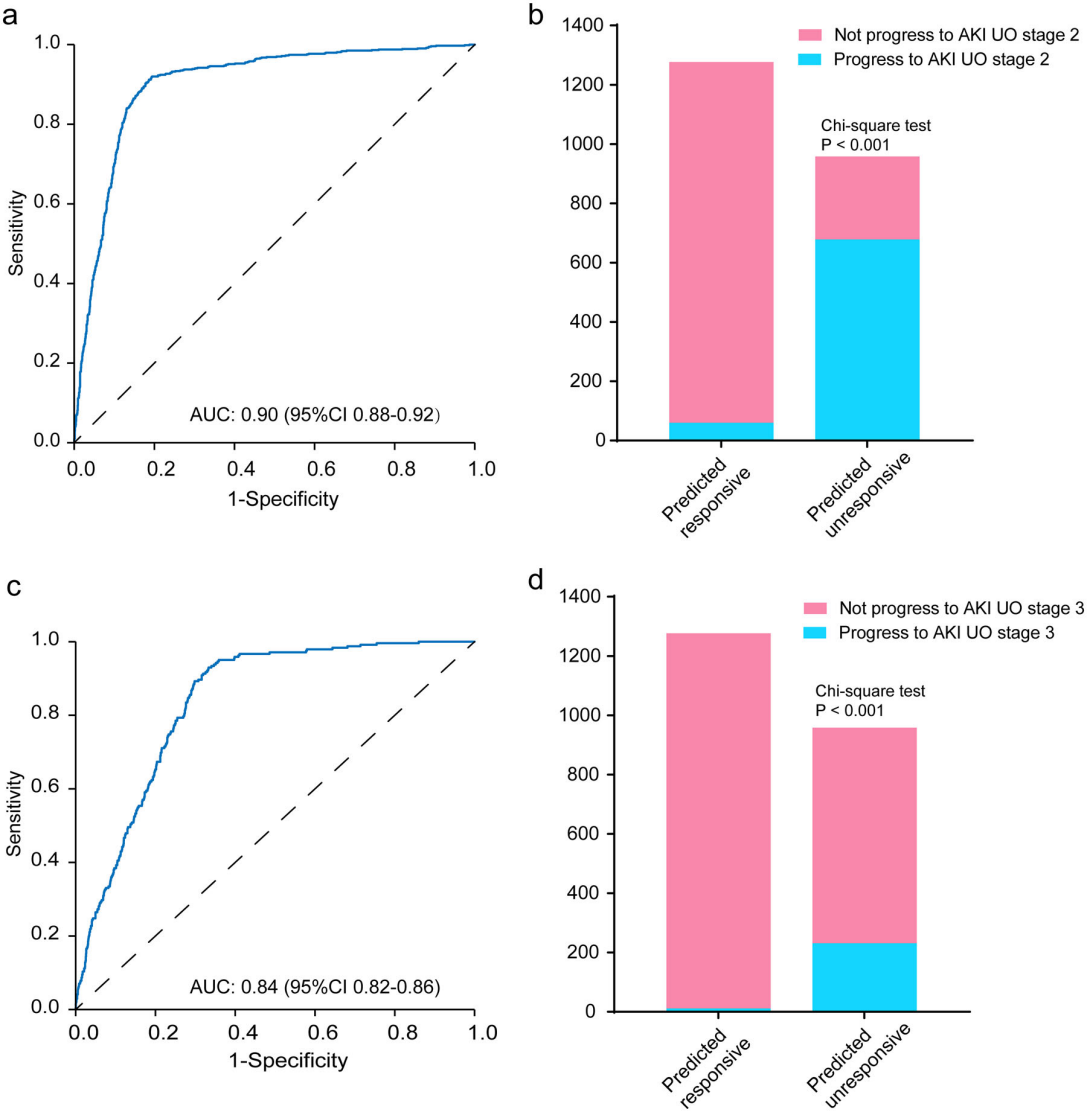
Performance of the XGBoost model in predicting AKI UO stage 1 progress to AKI UO stage 2 (a, b) and predicting AKI UO stage 1 progress to AKI UO stage 3 (C, D). The left panels present the ROC analyses for the model. The right panels show the risk-classification performance of the model. UO: urine output; AKI: acute kidney injury.

Furosemide is actively secreted by the renal proximal tubules into the lumen where it interacts with the Na+/K+/2Cl co-transporters. A good diuretic response to furosemide could be considered as a ‘proxy’ for having some residual renal function. This is the rationale that furosemide stress test was proposed to predict the progression of AKI [35]. Because indiscriminate use of loop diuretics can be harmful, appropriate patient selection prior to furosemide administration is mandatory. Using the ML model, we can easily triage patients to different subsets, and provide reliable reference on whether furosemide would be effective. The model achieved a PPV of 96.55% and NPV of 89.64% for furosemide responsiveness assessment, implies that it has a high prediction power for this task. Furthermore, when used the predicted FU probability as AKI progression risk, we found that it had an AUC of 0.90 for predicting AKI UO stage 1 progress to AKI UO stage 2, and an AUC of 0.84 for predicting AKI UO stage 1 progress to AKI UO stage 3 (Figure 4(a,c)), which was comparable to the furosemide stress test that predict the AKI progression [35]. In addition, patients predicted as FU AKI were significantly more prone to progress to AKI UO stage 2 or 3 (Figure 4(b,d)). These results suggested that the furosemide responsiveness prediction model had a satisfied discriminant capacity to predict the progression risk of early stage oliguric AKI.

### Strengths and limitations

This study has some strengths and limitations. The XGBoost algorithm is a novel technique that has been widely adopted in patient outcomes prediction in recent years [13,14,36–38], which achieved higher predictive accuracy than generalized linear model. Another advantage is the interpretable model not just provides what the prediction is, but also explains why. In this way, we could detect which features would affect the diuretic effect of furosemide most for a specific patient and thus applied individualized treatment strategy. Furthermore, the XGBoost model performed well in the independent validation cohort, showed good promise for generalization.

A limitation of this study is that it was not a pre-designed clinical trial that the indications, dosage and timing of furosemide administration could be prespecified. Future randomized controlled trials with uniform furosemide administration strategy are warranted to further validate the model. Second, more than 95% of furosemide bound to serum albumin, which mediates its renal tubular secretion and the subsequent diuretic effect [39]. However, the high ratio of missing values hampered our ability to evaluate the albumin on diuretic effect prediction. Adding albumin level to our model may improve the prediction outcomes. Third, the study only explored the short-term effect of furosemide administration, other long-term outcomes such as persistent kidney injury, and organ-failure free days, were not investigated. However, we have demonstrated that the model can accurately predict the progression of AKI to higher stages, and patients predicted as FR had a significantly better 30-day survival. Fourth, we defined diuretic response as UO >0.65 mL/kg/h (30% elevation) after furosemide administration according to previous literature [13], which needs more evidence. It should be interpreted as exploratory, and the best thresholds should be validated in the future. Fifth, significant heterogeneity was observed between the training and validation cohort, which might impair the prediction accuracy of the model. Besides, the model may be overfitting, which need to be validated in a larger cohort to confirm its value in clinical decision-making. Finally, in terms of mechanism, loop diuretics are secreted into the proximal convoluted tubule and decrease the activity of the sodium-potassium-chloride cotransporter. If glomerular filtration stops, loop diuretics are completely ineffective, which explains why anuric patients do not benefit from the substances at all. Therefore, the model may exhibit limited predictive power for anuria patients.

## Conclusions

In conclusion, we successfully applied the ML method to predict furosemide responsiveness in patients with oliguric AKI. We demonstrated that the XGBoost modeling technique could identify the key predictors of FR AKI that were not apparent in logistic regression, resulting in a better-performing predictive model to triage patients. The use of new technological methods, such as ML, are not a substitute for clinicians currently, but they could effectively enhance clinical decision-making. Further clinical trials are necessary to evaluate the model’s utility within a clinical decision support system.

## Supplementary Material

Supplemental Material

## Data Availability

Deidentified data pertaining to specific analysis may be available upon reasonable request from the corresponding author, pending approval from Monash Health research directorate.
