# Supplementary material for: Explainable machine learning model for predicting furosemide responsiveness in patients with oliguric acute kidney injury
Source: Ren Fail. 2023 Jan 16;45(1):2151468. doi: 10.1080/0886022X.2022.2151468 (PMC9848233; doi:10.1080/0886022X.2022.2151468)
Supplement: Supplemental Material [file IRNF_A_2151468_SM9256.pdf]

Appendix 1 Details on machine learning models

XGBoost uses an ensemble of gradient-boosted decision trees, and has been widely applied within the domains of computer. The advantage of using a boosted ensemble algorithm is the fact that it can combine multiple weak classifiers to produce a single strong classifier, which can improve prediction modeling. While the XGBoost model often achieves higher accuracy than a single decision tree, it sacrifices the intrinsic interpretability of decision trees. For example, following the path that a decision tree takes to make its decision is trivial and self-explained, but following the paths of hundreds or thousands of trees is much harder. To achieve both performance and interpretability, some model compression techniques allow transforming an XGBoost into a single "born-again" decision tree that approximates the same decision function.

Support vector machines (SVM) are supervised learning models with associated learning algorithms that analyze data for classification and regression analysis. A support-vector machine constructs a hyperplane or set of hyperplanes in a high- or infinite-dimensional space, which can be used for classification, regression, or other tasks like outliers detection. Intuitively, a good separation is achieved by the hyperplane that has the largest distance to the nearest training-data point of any class (so-called functional margin), since in general the larger the margin, the lower the generalization error of the classifier.

K-Nearest Neighbour (KNN) is a non-parametric supervised learning method first developed by Evelyn Fix and Joseph Hodges in 1951, and later expanded by Thomas Cover. It is used for classification and regression. In both cases, the input consists of the k closest training examples in a data set. The output depends on whether k-NN is used for classification or regression:

In k-NN classification, the output is a class membership. An object is classified by a plurality vote of its neighbors, with the object being assigned to the class most common among its k nearest neighbors (k is a positive integer, typically small). If k = 1, then the object is simply assigned to the class of that single nearest neighbor.

In k-NN regression, the output is the property value for the object. This value is the average of the values of k nearest neighbors.

k-NN is a type of classification where the function is only approximated locally and all computation is deferred until function evaluation. Since this algorithm relies on distance for classification, if the features

Information Classification: General

represent different physical units or come in vastly different scales then normalizing the training data can improve its accuracy dramatically.

Both for classification and regression, a useful technique can be to assign weights to the contributions of the neighbors, so that the nearer neighbors contribute more to the average than the more distant ones. For example, a common weighting scheme consists in giving each neighbor a weight of 1/d, where d is the distance to the neighbor.

The neighbors are taken from a set of objects for which the class (for k-NN classification) or the object property value (for k-NN regression) is known. This can be thought of as the training set for the algorithm, though no explicit training step is required.

Random forest is an ensemble learning method for classification, regression and other tasks that operates by constructing a multitude of decision trees at training time. For classification tasks, the output of the random forest is the class selected by most trees. For regression tasks, the mean or average prediction of the individual trees is returned. Random decision forests correct for decision trees' habit of overfitting to their training set. Random forests generally outperform decision trees, but their accuracy is lower than gradient boosted trees. However, data characteristics can affect their performance

Information Classification: General

Table S1 The missing value distribution on MIMIC-IV and eICU-CRD data.

a. MIMIC-IV

Variables	Number of missing values	Percent
aniongap_min	1,215	17.6%
aniongap_max	1,215	17.6%
bicarbonat_min	923	13.4%
bicarbonat_max	923	13.4%
creatinine_min	895	13.0%
creatinine_max	895	13.0%
chloride_min	582	8.4%
chloride_max	582	8.4%
glucose_min	463	6.7%
glucose_max	463	6.7%
hematocrit_min	798	11.6%
hematocrit_max	798	11.6%
hemoglobin_min	835	12.1%
hemoglobin_max	835	12.1%
lactate_min	1,386	20.1%
lactate_max	1,386	20.1%
platelet_min	957	13.9%
platelet_max	957	13.9%
potassium_min	534	7.7%
potassium_max	534	7.7%
aptt_min	1,377	19.9%
aptt_max	1,377	19.9%
inr_min	1,365	19.7%
inr_max	1,365	19.7%
pt_min	1,348	19.5%
pt_max	1,348	19.5%
sodium_min	578	8.4%
sodium_max	578	8.4%
bun_min	905	13.1%
bun_max	905	13.1%
wbc_min	973	14.1%
wbc_max	973	14.1%
heartrate_min	235	3.4%
heartrate_max	235	3.4%
heartrate_mean	235	3.4%
sysbp_min	283	4.1%
sysbp_max	283	4.1%
sysbp_mean	283	4.1%
diasbp_min	283	4.1%
diasbp_max	283	4.1%
diasbp_mean	283	4.1%
meanbp_min	233	3.4%
meanbp_max	233	3.4%
meanbp_mean	233	3.4%

Information Classification: General

resprate_min	208	3.00%
resprate_max	208	3.00%
resprate_mean	208	3.00%
tempc_min	1,016	14.70%
tempc_max	1,016	14.70%
tempc_mean	1,016	14.70%
spo2_min	252	3.70%
spo2_max	252	3.70%
spo2_mean	252	3.70%
glucose_mean	463	6.70%

b**. eICU-CRD**

**Variable**	**Number of missing values**	**Percent**
aniongap_min	437	19.6%
aniongap_max	437	19.6%
Bicarbonate_min	157	7.0%
Bicarbonate_max	157	7.0%
Creatinine_min	142	6.4%
Creatinine_max	142	6.4%
chloride_min	88	3.9%
chloride_max	88	3.9%
glucose_min	220	9.8%
glucose_max	220	9.8%
Hematocrit_min	117	5.2%
Hematocrit_max	117	5.2%
Hemoglobin_min	128	5.7%
Hemoglobin_max	128	5.7%
lactate_min	435	19.5%
lactate_max	435	19.5%
platelet_min	152	6.8%
platelet_max	152	6.8%
potassium_min	78	3.5%
potassium_max	78	3.5%
ptt_min	309	13.8%
ptt_max	309	13.8%
inr_min	305	13.6%
inr_max	305	13.6%
pt_min	305	13.6%
pt_max	305	13.6%
sodium_min	86	3.8%
sodium_max	86	3.8%
bun_min	146	6.5%
bun_max	146	6.5%
wbc_min	155	6.9%
wbc_max	155	6.9%

Information Classification: General

Heartrate_min	73	3.3%
heartrate__max	73	3.3%
Heartrate_mean	73	3.3%
sysbp_min	83	3.7%
sysbp_max	83	3.7%
sysbp_mean	83	3.7%
diasbp_min	83	3.7%
diasbp_max	83	3.7%
diasbp_mean	83	3.7%
meanbp_min	72	3.2%
meanbp_max	72	3.2%
meanbp_mean	72	3.2%
resprate_min	69	3.1%
resprate_max	69	3.1%
resprate_mean	69	3.1%
tempc_min	418	18.7%
tempc_max	418	18.7%
tempc_mean	418	18.7%
spo2_min	78	3.5%
spo2_max	78	3.5%
spo2_mean	78	3.5%
glucose_mean	220	9.8%

Information Classification: General

Table S2 Major packages of R and python software used in this study

a. Packages of R software

**Functions**	**R package**
Multivariate logistic regression analysis	glm
Plot the receiver operating curve (ROC) and measure the area under the ROC (AUC)	pROC
For ROC analysis to determine optimal cutoff value	OptimalCutpoints
Plot bar diagrams	ggplot2
Plot calibration curves	rms
Decision curve analysis (DCA)	rmda

b. Packages of python software

**Functions**	**Python package**
For training the XGBoost model	xgboost
Calculating the SHAP value of specific feature	shap
For training the random forest model	sklearn.ensemble
For training the SVM model	sklearn.svm
For training the KNN model	sklearn.neighbors
Plot precision-Recall curve	sklearn.metrics

Information Classification: General

Table S3 Characteristics between the training and validation groups

Variables	Training set (MIMIC-IV)	Validation set (eICU-CRD)	P value	SMD
Patient population, n	6897	2235		
Age (SD)	70.0 (12.9)	70.4 (13.0)	0.167	0.034
Male (%)	4024 (58.3)	1238 (55.4)	0.015	0.06
Total fluid intake between 6-24 h (median [IQR])	3345.0 [1397.2, 6210.0]	3740.0 [1475.0, 7292.5]	<0.001	0.158
Comorbid illness				
Congestive heart failure (%)	3338 (48.4)	418 (18.7)	<0.001	0.663
Chronic kidney disease (%)	1837 (26.6)	519 (23.2)	0.001	0.079
Severe liver disease (%)	250 (3.6)	134 (6.0)	<0.001	0.111
Total furosemide administration between 6-12 h (median [IQR])	60.0 [40, 80]	45.0 [40, 60]	0.01	0.002
Length of ICU stay, day (SD)	5.2 (6.3)	4.8 (5.7)	0.024	0.056
Inhospital mortality (%)	841 (12.2)	239 (10.7)	0.061	0.047
Furosemide responsive (%)	3981 (57.7)	1334 (59.7)	0.107	0.04
Ethnicity (%)			<0.001	0.126
African American	548 (7.9)	148 (6.6)		
White	4987 (72.3)	1737 (77.7)		
Other	1362 (19.7)	350 (15.7)		
Admission type (%)			<0.001	0.786
Elective	1868 (27.1)	645 (28.9)		
Emergency	3244 (47.0)	1561 (69.8)		
Urgent	1785 (25.9)	29 (1.3)		
Vasopressor use, n (%)	2518 (36.5)	749 (33.5)	0.011	0.063
Infection, n (%)	1879 (27.2)	1013 (45.3)	<0.001	0.383
Mechanical ventilation, n (%)	2681 (38.9)	1176 (52.6)	<0.001	0.279
Minimum bicarbonate (mmol/l, median [IQR])	23.0 [21.0, 25.0]	24.0 [22.0, 26.0]	<0.001	0.211
Maximum bicarbonate (mmol/l, median [IQR])	24.0 [21.0, 26.0]	24.0 [22.0, 27.0]	<0.001	0.243
Minimum creatinine (mg/dL, median [IQR])	1.0 [0.8, 1.5]	1.0 [0.8, 1.4]	0.229	0.039
Maximum creatinine (mg/dL, median [IQR])	1.0 [0.8, 1.5]	1.0 [0.8, 1.5]	0.367	0.023
Minimum chloride (mmol/l)	102.0 (5.7)	101.8 (5.6)	0.19	0.033
Maximum chloride (mmol/l)	105.4 (6.7)	106.1 (6.8)	<0.001	0.107
Minimum glucose (mg/dL, median [IQR])	122.0 [104.1, 150.0]	119.0 [102.0, 145.0]	<0.001	0.057
Maximum glucose (mg/dL, median [IQR])	166.0 [135.0, 200.0]	161.0 [132.0, 193.0]	0.001	0.07
Minimum hematocrit (%)	28.4 (6.7)	28.2 (6.8)	0.337	0.024
Maximum hematocrit (%)	34.8 (6.3)	34.9 (5.9)	0.482	0.018
Minimum hemoglobin (g/dL)	9.3 (2.2)	9.4 (2.2)	0.263	0.028
Maximum hemoglobin (g/dL)	11.4 (2.2)	11.6 (2.0)	0.023	0.059
Minimum lactate (mmol/L)	1.7 (1.2)	1.6 (1.1)	0.005	0.077
Maximum lactate (mmol/L)	6.1 (7.1)	6.0 (7.1)	0.47	0.018
Minimum platelet ($\times 10^9$/l, median [IQR])	159.0 [118.0, 216.0]	178.0 [132.0, 239.0]	<0.001	0.203
Maximum platelet ($\times 10^9$/l, median [IQR])	177.0 [136.0, 234.0]	198.0 [153.0, 263.0]	<0.001	0.233
Minimum potassium (mmol/l)	4.0 (0.6)	4.0 (0.6)	0.006	0.069

Information Classification: General

Maximum potassium (mmol/l)	4.9 (0.9)	4.9 (1.0)	0.061	0.046
Minimum aPTT (s)	35.8 (19.1)	35.3 (17.2)	0.253	0.031
Maximum aPTT (s)	42.2 (25.5)	41.7 (24.9)	0.424	0.021
Minimum INR	1.5 (0.8)	1.5 (0.9)	0.08	0.045
Maximum INR	1.6 (0.9)	1.7 (1.1)	0.125	0.039
Minimum PT (s)	16.1 (8.4)	16.5 (7.8)	0.027	0.059
Maximum PT (s)	17.7 (9.7)	18.0 (9.9)	0.134	0.039
Minimum sodium (mmol/l)	135.9 (4.7)	136.4 (4.6)	<0.001	0.099
Maximum sodium (mmol/l)	138.5 (4.5)	139.0 (4.5)	<0.001	0.123
Minimum BUN (mg/dl, median [IQR])	21.0 [15.0, 33.0]	21.0 [15.0, 33.0]	0.229	0.018
Maximum BUN (mg/dl, median [IQR])	21.0 [15.0, 34.0]	22.0 [16.0, 35.0]	0.099	0.027
Minimum WBC ($\times 10^9$/l)	12.1 (7.8)	11.6 (7.7)	0.014	0.063
Maximum WBC ($\times 10^9$/l)	19.2 (13.9)	15.7 (11.1)	<0.001	0.281
Minimum heartrate (/min)	75.8 (15.7)	76.2 (15.6)	0.319	0.025
Maximum heartrate (/min)	93.4 (18.9)	93.2 (17.8)	0.687	0.01
Minimum systolic BP (mmHg)	96.8 (17.4)	97.9 (18.1)	0.012	0.062
Maximum systolic BP (mmHg D)	135.1 (21.6)	137.1 (22.0)	<0.001	0.09
Minimum diastolic BP (mmHg)	50.2 (11.0)	49.3 (11.4)	0.001	0.08
Maximum diastolic BP (mmHg)	75.2 (17.9)	74.2 (16.8)	0.025	0.057
Minimum mean BP (mmHg)	63.5 (12.8)	62.6 (13.5)	0.007	0.066
Maximum mean BP (mmHg)	93.6 (21.5)	93.7 (22.5)	0.918	0.003
Minimum respiratory rate (/min)	14.4 (4.3)	14.2 (4.4)	0.026	0.055
Maximum respiratory rate (/min)	24.1 (6.8)	23.8 (6.7)	0.041	0.051
Minimum temperature (°C)	36.3 (0.8)	36.2 (0.8)	<0.001	0.16
Maximum temperature (°C)	36.8 (0.7)	36.7 (0.8)	0.001	0.083
Minimum Spo2 (%)	94.4 (5.8)	94.3 (6.1)	0.58	0.014
Maximum Spo2 (%)	99.2 (1.8)	99.1 (1.8)	0.42	0.02

SD, standard derivation; IQR, interquartile range; BP, Blood pressure; BUN, blood urea nitrogen; SMD, standardized mean difference.

Information Classification: General

Table S4 Multivariable logistic regression model with stepwise variable selection

**Variables**	**OR (95% CI)**	**P value**
Mechanical ventilation	0.78 (0.69-0.87)	0.000
Maximum creatinine	0.26 (0.11-0.60)	0.002
Maximum hematocrit	0.93 (0.90-0.97)	0.000
Minimum hemoglobin	0.93 (0.89-0.97)	0.001
Maximum hemoglobin	1.34 (1.19-1.50)	0.000
Minimum lactate	0.89 (0.84-0.94)	0.000
Minimum platelet	1.00 (1.00-1.01)	0.015
Maximum platelet	1.00 (1.00-1.00)	0.010
Minimum potassium	0.74 (0.66-0.84)	0.000
Maximum potassium	1.17 (1.08-1.28)	0.000
Minimum BUN	0.95 (0.91-1.00)	0.050
Maximum BUN	1.05 (1.00-1.11)	0.038
Minimum WBC	0.99 (0.98-1.00)	0.009
Maximum heartrate	1.01 (1.00-1.01)	0.001
Minimum systolic BP	1.01 (1.00-1.01)	0.000
Maximum systolic BP	1.01 (1.00-1.01)	0.000
Minimum diastolic BP	1.01 (1.00-1.01)	0.018
Maximum respiratory rate	1.01 (1.00-1.02)	0.009
Admission type		
Elective (ref.)		
Emergency	1.05 (0.91-1.21)	0.500
Urgent	1.21 (1.05-1.40)	0.011

Backward logistic regression was performed with 5% significance level, at each iteration of backward logistic regression the variable with highest p-value was removed until all variables had at least p-value less than or equal to 0.05.

OR, odds ratio; BP, Blood pressure; BUN, blood urea nitrogen.

Information Classification: General

Table S5 Parameter Grid explored for XGBoost model

**Parameter**	**Grid values**
Learning Rate	0.005,0.01,0.02, 0.05,0.1
Max Depth	3,5,7,9
Min Child Weight	1,3,5
Subsample	0.5, 0.55, 0.6, 0.65, 0.7,0.75, 0.8
Column sample	0.5, 0.55, 0.6, 0.65, 0.7, 0.75
Number estimators	50, 100, 250, 500, 1000
Gamma	0, 0.1,0.2,0.3,0.4,0.5

Final optimal model parameters

Parameter	Value
Learning Rate	0.05
Max Depth	6
Min Child Weight	1
Subsample	1
Column sample	1
Number estimators	1000
Gamma	0

Information Classification: General

Table S6 Performance metrics of the machine learning models in the training and validation cohort after removing missing data

a. Training cohort

	**AUC**	**Accuracy**	**Sensitivity**	**Specificity**	**PPV**	**NPV**	**Brier score**	**Kappa***
Logistic regression	0.62 (0.59-0.65)	63.1% (60.15-65.96%)	66.46% (62.69-70.03%)	58.12% (53.37-62.72%)	70.23% (66.45-73.76%)	53.81% (49.24-58.32%)	0.233	0.24
XGBoost	0.96 (0.93-0.97)	95.84% (94.46-96.88%)	94.79% (92.77-96.26%)	97.41% (95.41-98.55%)	98.20% (96.81-98.99%)	96.20% (89.79-94.68%)	0.052	0.91
SVM	0.95 (0.92-0.96)	95.74% (94.35-96.80%)	96.47% (94.26-97.85%)	95.25% (93.3-96.66%)	93.18% (90.43-95.18%)	97.57% (96.03-98.52%)	0.103	0.91
KNN	0.64 (0.61-0.67)	65.37% (62.45-68.18%)	69.94% (66.25-73.38%)	68.59% (53.85-63.17%)	71.52% (67.84-74.94%)	56.72% (52.05-61.28%)	0.165	0.28
Random Forrest	0.98 (0.97-0.99)	98.11% (97.10-98.77%)	97.63% (96.12-98.56%)	98.82% (97.28-99.50%)	99.20% (98.13-99.66%)	96.55% (94.39-97.90%)	0.076	0.96

b. validation cohort

	**AUC**	**Accuracy**	**Sensitivity**	**Specificity**	**PPV**	**NPV**	**Brier score**	**Kappa***
Logistic regression	0.62 (0.59-0.65)	62.39% (59.51-61.59%)	64.33% (60.59-67.9%)	59.61% (55.05-64.00%)	69.52% (65.75-73.05%)	53.85% (49.49-58.14%)	0.231	0.24
XGBoost	0.94 (0.93-0.96)	93.9% (92.33-95.16%)	91.92% (89.58-93.77%)	96.72% (94.67-98.01%)	97.57% (96.03-98.52%)	89.31% (86.29-91.74%)	0.041	0.88
SVM	0.83 (0.80-0.85)	63.81% (59.57-67.85%)	53.57% (40.7-65.98%)	65.07% (60.59-69.29%)	15.79% (11.29-21.65%)	91.98% (88.5-94.47%)	0.132	0.15
KNN	0.77 (0.75-0.79)	73.34% (70.67-75.85%)	57.47% (53.65-61.2%)	96.07% (93.87-97.50%)	95.44% (92.91-97.1%)	61.2% (57.58-64.69%)	0.163	0.49
Random Forrest	0.91 (0.89-0.92)	90.93% (89.1-92.48%)	91.92% (89.58-93.77%)	89.52% (86.38-92.0%)	92.63% (90.36-94.39%)	88.55% (85.33-91.14%)	0.088	0.81

Unless otherwise indicated, data are percentages, with 95% confidence intervals in brackets.

*Measures the agreement between the prediction and the ground truth.

PPV, Positive Predictive Value; NPV, Negative Predictive Value

Information Classification: General

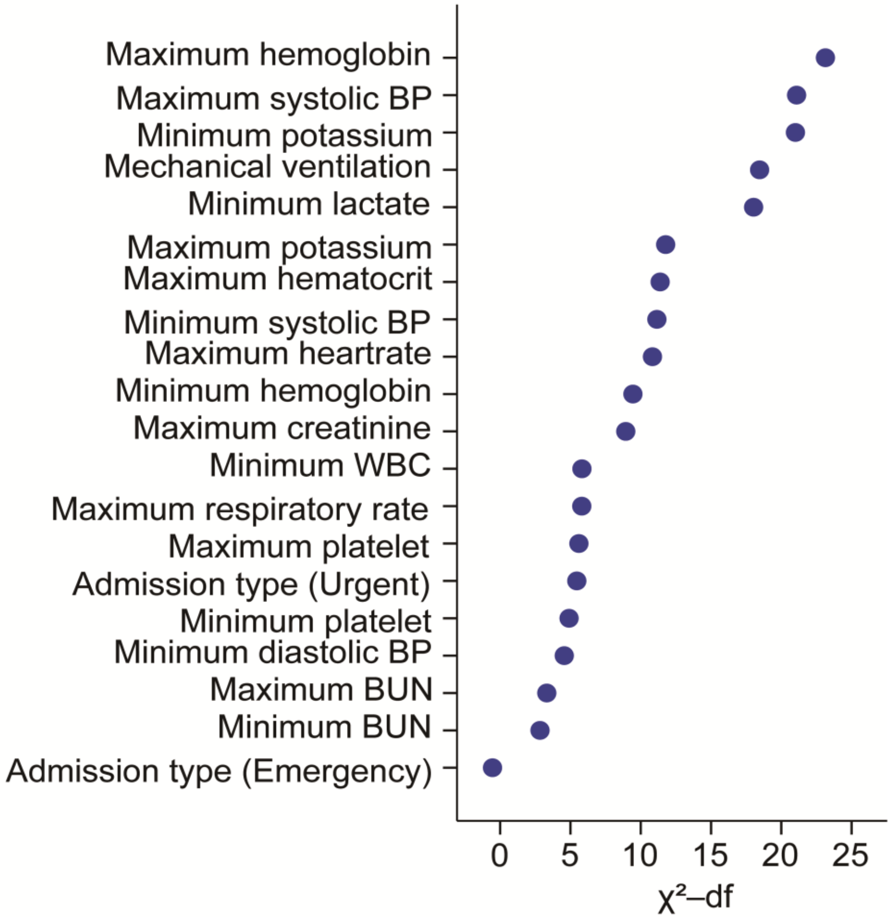

Fig. S1 Importance of each variable in the logistic model as measured by partial Wald χ^2 minus the predictor degrees of freedom. BP, Blood pressure; BUN, blood urea nitrogen.

Information Classification: General

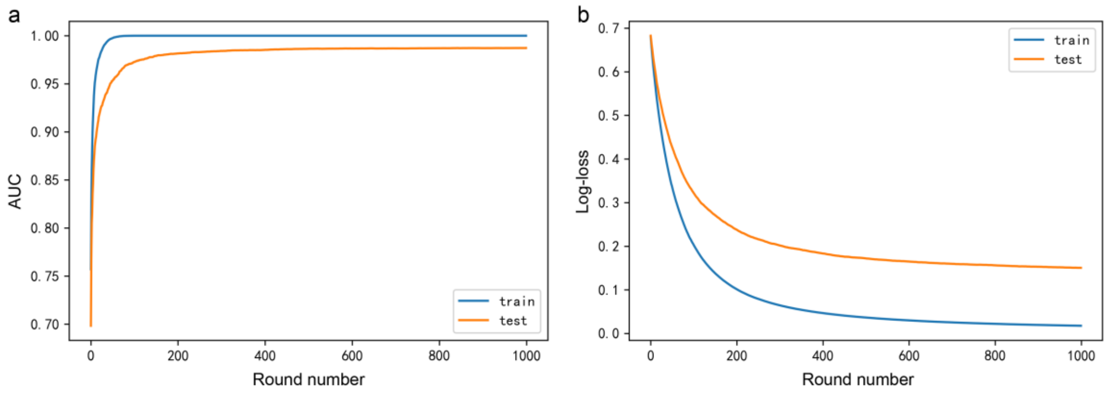

Fig. S2 Training process of the XGBoost model. Rising curves (A) represent AUC of training and validation sets, and falling curves (B) represent log-loss of training and validation sets, indicating fit between prediction and truth label.

Information Classification: General

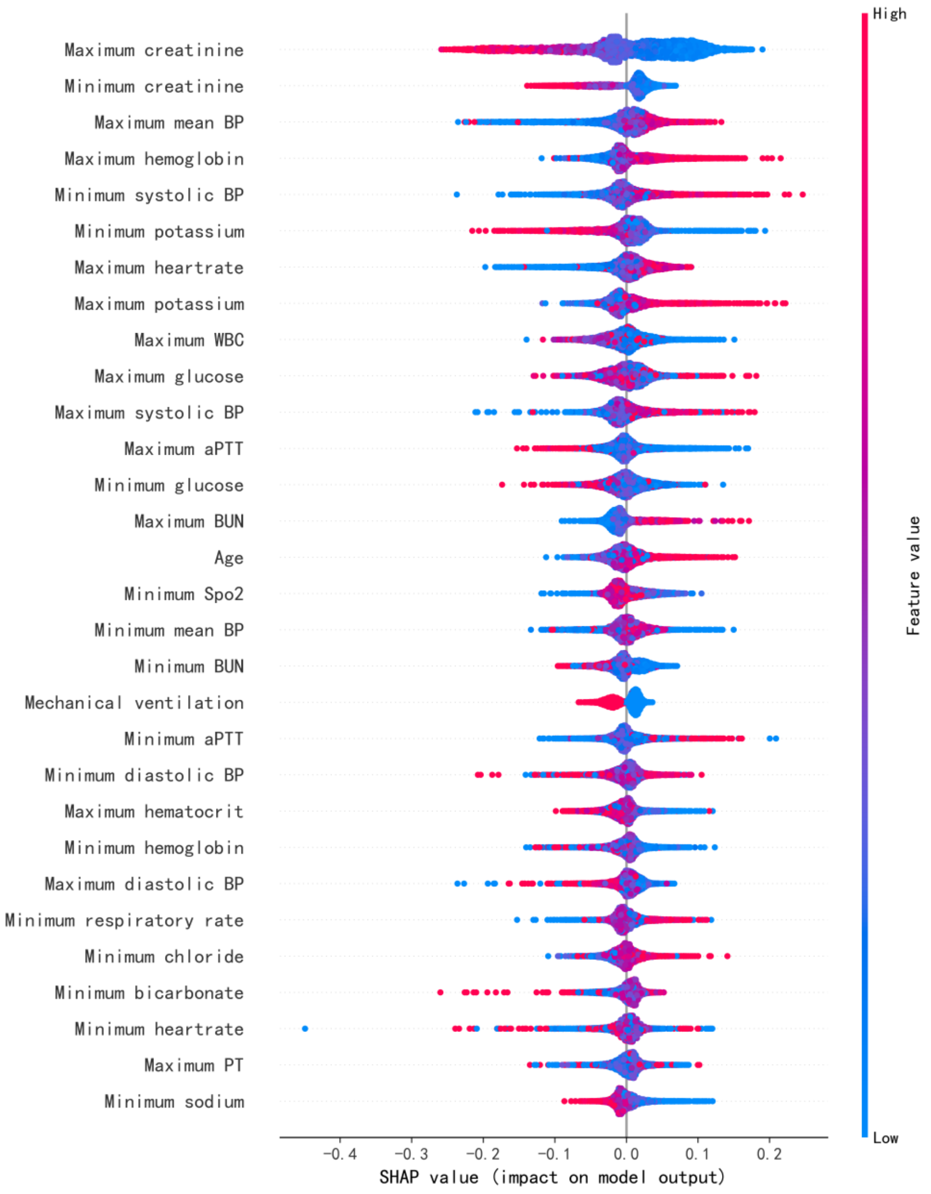

Fig. S3 SHAP summary plot of the top 30 features of the XGBoost model. The plot sorts features by the sum of SHAP value magnitudes over all samples. The colour represents the feature value (red high, blue low). The x axis measures the impact on the model output (right positive, left negative). BP, blood pressure; WBC, white blood count; BUN, blood urea nitrogen.

Information Classification: General

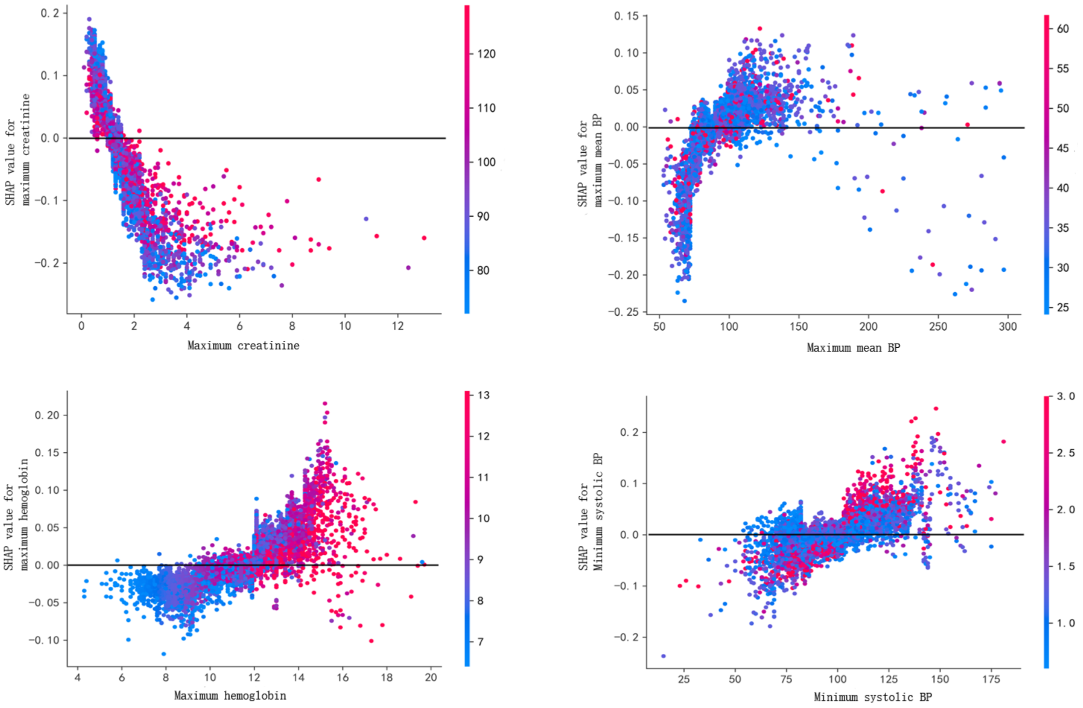

Fig. S4 SHAP dependence plot of the XGBoost model. The SHAP dependence plot shows how a single feature affects the output. SHAP values for specific features exceed zero, representing an increased probability of furosemide responsiveness. The variables of maximum creatinine, maximum mean BP, maximum hemoglobin and minimum systolic BP were shown. BP, blood pressure.

Information Classification: General

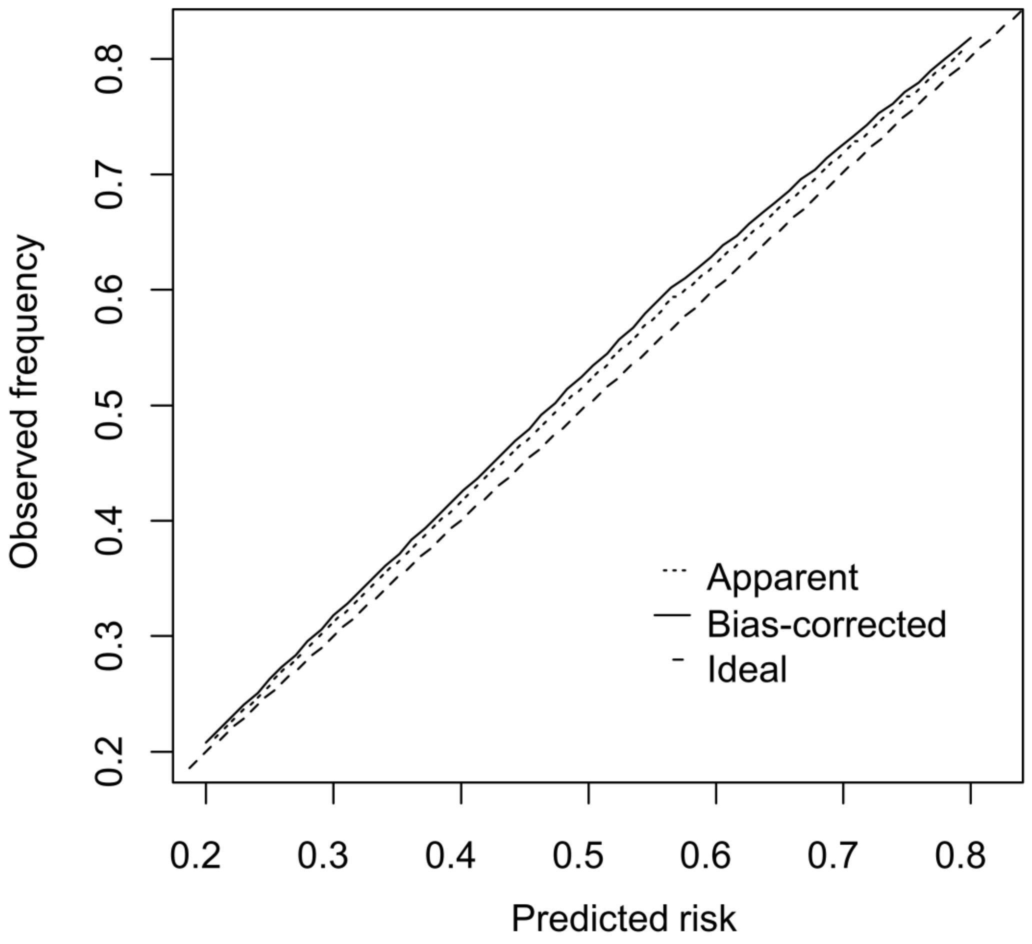

Fig. S5 Calibration curve of the XGBoost model in the test set. The Brier score of the model was 0.043.

Information Classification: General

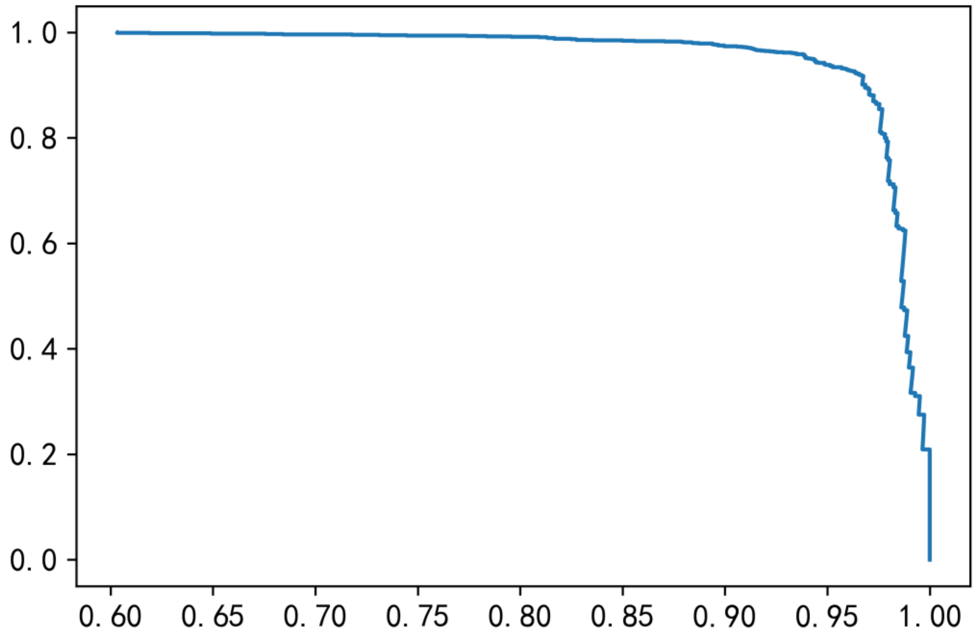

Fig. S6 Precision-Recall curve for the XGBoost model (Validation Set).

Information Classification: General

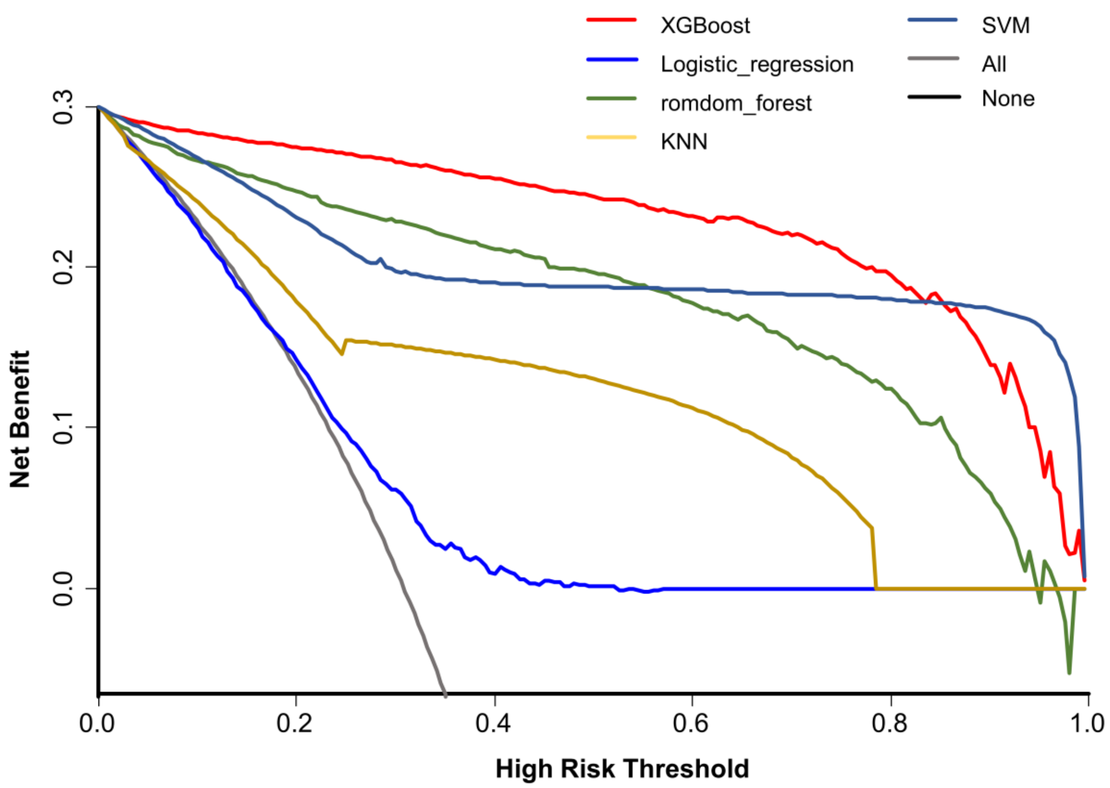

Fig. S7 Net decision curves for the XGBoost model and other machine learning models (Validation Set).

Information Classification: General

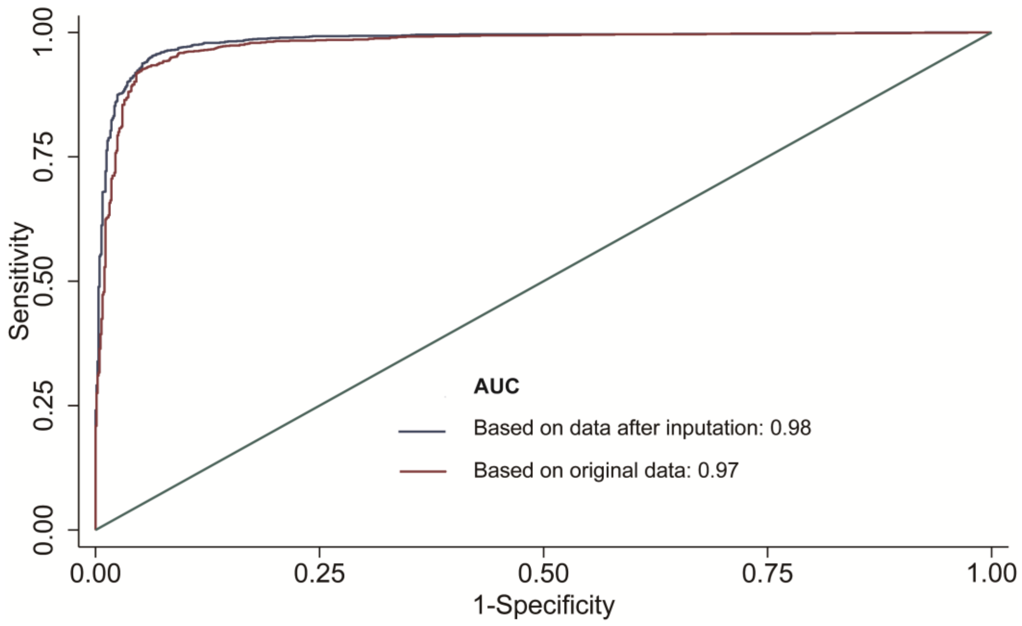

Fig. S8 ROC curves for the XGBoost model based on data before and after single imputation for missing values (Validation Set).

Information Classification: General
